# Non-High-Density Lipoprotein Cholesterol and Progression of Chronic Kidney Disease: Results from the KNOW-CKD Study

**DOI:** 10.3390/nu14214704

**Published:** 2022-11-07

**Authors:** Sang Heon Suh, Tae Ryom Oh, Hong Sang Choi, Chang Seong Kim, Eun Hui Bae, Seong Kwon Ma, Kook-Hwan Oh, Young Youl Hyun, Suah Sung, Soo Wan Kim

**Affiliations:** 1 Department of Internal Medicine, Chonnam National University Medical School and Chonnam National University Hospital, Gwangju 61469, Korea; 2 Department of Internal Medicine, Seoul National University Hospital, Seoul 03080, Korea; 3 Department of Internal Medicine, Kangbuk Samsung Hospital, Sungkyunkwan University School of Medicine, Seoul 03181, Korea; 4 Department of Internal Medicine, Eulji Medical Center, Eulji University, Seoul 01830, Korea

**Keywords:** chronic kidney disease, estimated glomerular filtration rate, non-high-density lipoprotein cholesterol, renal outcome

## Abstract

As the relation between serum non-high-density lipoprotein cholesterol (nHDL) level and renal outcomes has never been investigated in patients with non-dialysis chronic kidney disease (CKD) yet, we here aimed to unveil the association of nHDL with CKD progression. A total of 2152 patients with non-dialysis CKD at stages 1 to 5 from the KNOW-CKD study were categorized into the tertile (i.e., 1st (T1), 2nd (T2), and 3rd (T3) tertiles) by nHDL, and were prospectively analyzed. The primary outcome was the composite renal event, defined as a composite of decline of kidney function or onset of end-stage renal disease. Kaplan–Meier survival curves analysis demonstrated that the cumulative incidence of the composite renal event was significantly increased in T1 and T3, compared to T2 (*p* = 0.028, by Log-rank test). Cox regression analysis revealed that both T1 (adjusted hazard ratio 1.309, 95% confidence interval 1.074–1.595) and T3 (adjusted hazard ratio 1.272, 95% confidence interval 1.040–1.556) are associated with significantly increased risk of a composite renal event, compared to T2. The restricted cubic spline plot demonstrated a non-linear, U-shaped association between nHDL and the risk of a composite renal event. In conclusion, both low and high serum nHDL levels are associated with increased risk of CKD progression.

## 1. Introduction

Dyslipidemia is a common complication of chronic kidney disease (CKD) [1,2,3]. The current guidelines on the management of dyslipidemia in CKD recommends the use of statins, which mainly target low-density lipoprotein cholesterol (LDL-C) levels to prevent cardiovascular (CV) events [4]. Yet, among the patients with CKD, abnormalities in triglycerides (TG) and high-density lipoprotein cholesterol (HDL-C) levels are more prominent characteristics [1,2,3], which are known to increase the risk of adverse CV events in the general population [5,6,7] as well as in patients with CKD [8,9]. Thus, it seems obvious that dyslipidemia is a therapeutic target of paramount importance in the management of CKD.

Mounting evidence now suggests that dyslipidemia is not only a complication of CKD, but may also be associated with the development and progression of CKD [10,11,12,13,14], as shown in a number of large-scale, prospective cohort studies. The Physicians’ Health Study demonstrated that a variety of abnormal lipid profiles including high non-high-density lipoprotein cholesterol (nHDL) were closely related to the risk of renal dysfunction in 4483 initially healthy subjects [15]. The Framingham Offspring Study reported that, in the analysis of 2585 participants, the HDL-C level reduced with the risk of incident CKD [16]. By analyzing 12,728 subjects, the Atherosclerosis Risk in Communities Study concluded that high TG and low HDL-C, but not LDL-C, levels are associated with the risk of renal dysfunction [17]. Although numerous lipid indices have been examined so far, there is still no conclusive result that one is superior to the other in the estimation of renal prognosis among the patients with non-dialysis CKD.

The serum nHDL level is equal to total cholesterol minus HDL-C [18], and represents all atherogenic lipoproteins, including intermediate-density lipoprotein, lipoprotein(a), LDL-C, and very low-density lipoprotein remnants [19], indicating that nHDL may be associated with adverse CV outcomes. Indeed, we recently reported that the elevated serum level of nHDL increased the risk of composite CV events among the patients with CKD [20]. To the best of our knowledge, the relation between nHDL and CKD progression, however, has never been investigated yet.

Therefore, we hypothesized that an elevated serum level of nHDL may also be associated with an increased risk of CKD progression. Taking advantage of more than 2000 subjects from the Korean Cohort Study for Outcomes in Patients With Chronic Kidney Disease (KNOW-CKD) cohort, we here aimed to address the association of nHDL with renal outcomes in patients with non-dialysis CKD.

## 2. Materials and Methods

### 2.1. Study Design

**Figure 1:**
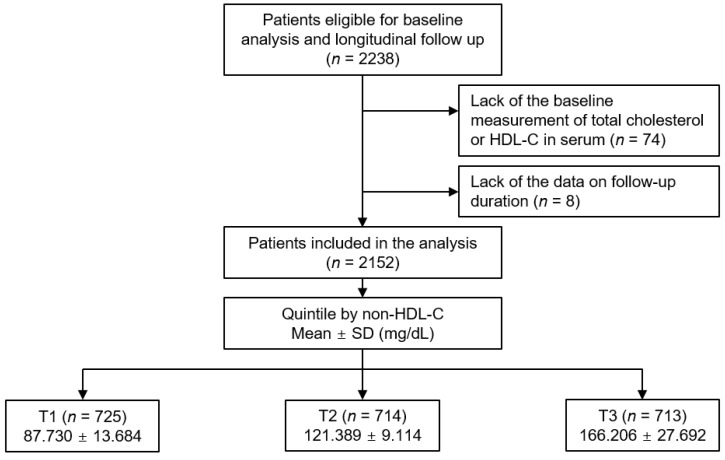
Study design. Abbreviations: HDL-C, high-density lipoprotein cholesterol; nHDL, non-high-density lipoprotein cholesterol; T1, 1st tertile; T2, 2nd tertile; T3, 3rd tertile; SD, standard deviation.

The KNOW-CKD study is previously described [21]. The study was conducted in accordance with the principles of the Declaration of Helsinki. The Institutional Review Board at each participating center approved the study protocol (Seoul National University Hospital (1104–089-359), Seoul National University Bundang Hospital (B-1106/129–008), Yonsei University Severance Hospital (4–2011-0163), Kangbuk Samsung Medical Center (2011–01-076), Seoul St. Mary’s Hospital (KC11OIMI0441), Gil Hospital (GIRBA2553), Eulji General Hospital (201105–01), Chonnam National University Hospital (CNUH-2011-092), and Busan Paik Hospital (11–091). Patients with non-dialysis CKD at all stages between the ages of 20 and 75 years were enrolled from 2011 through 2016. Informed consent was voluntarily obtained from all the participants. All the participants were under close observation during the follow-up period, and each participating center recorded the study outcomes. Among the participants who are longitudinally followed up (*n* = 2238), those without the baseline measurement of total cholesterol or HDL-C (*n* = 74), and those without the information on follow-up duration (*n* = 8), were excluded (Figure 1). Finally, a total of 2152 patients were included and analyzed. The study observation period ended on 31 March 2021. The median duration of follow-up was 6.940 years.

### 2.2. Data Collection from Participants

Demographic information included age, gender, Charlson comorbidity index, primary cause of CKD, smoking history, and medication history. Anthropometric measurements included body mass index (BMI) and systolic and diastolic blood pressures (SBP and DBP). After overnight fasting, venous samples were drawn to determine the baseline laboratory measurement, including lipid profiles. The serum nHDL level was defined as total cholesterol minus HDL-C. The estimated glomerular filtration rate (eGFR) was calculated by the Chronic Kidney Disease Epidemiology Collaboration equation using the serum creatinine level [22]. CKD stages were determined by the Kidney Disease Improving Global Outcomes (KDIGO) guidelines [23]. Albuminuria was measured in spot urine samples. Echocardiographic data were collected using complete two-dimensional M-mode and Doppler studies via standard approaches [24]. The cardiologists who are were blinded to the clinical data performed the echocardiography at each participating study site.

### 2.3. Exposure and Study Outcome

The exposure was nHDL, by which the subjects were categorized into the tertile (i.e., 1st (T1), 2nd (T2), and 3rd (T3) tertiles) (Figure 1). The primary outcome was the composite renal event, defined as a composite of decline of renal function (the first occurrence of > 50% decline of eGFR or doubling of serum creatinine from the baseline) or initiation of renal replacement therapy (RRT) during follow-up periods. The secondary outcome was each of decline of renal function and initiation of RRT. For the accuracy on the clinical outcomes, the participating investigators cross-checked all outcome events.

### 2.4. Statistical Analysis

In the comparison of the baseline characteristics by nHDL, continuous variates were analyzed by one-way analysis of variance, while categorical variates were analyzed by χ^2^ test. Cumulative incidences of outcome events were visualized using Kaplan–Meier curves. The participants with any missing data were excluded for further analyses. To evaluate independent associations between nHDL and study outcomes, Cox regression analyses were adopted. Patients lost to follow-up were censored at the date of the last visit. The results of Cox proportional hazard models were presented as hazard ratios (HRs) and 95% confidence intervals (CIs). Restricted cubic spline plots were used to assess the relation between nHDL (as a continuous variable) with the study outcomes. To confirm our findings, we performed sensitivity analyses. First, we exclude the participants with eGFR ≥ 90 mL/min/1.73 m^2^, because the CKD patients at stage 1 have nearly normal kidney function and were considered not to represent the patients with CKD well. Second, we excluded the subjects with eGFR < 15 mL/min/1.73 m^2^, because the CKD patients at stage 5, which were relatively scanty, and because the burden of CKD may overly impact the association between serum nHDL and study outcomes. Third, we evaluated the cause-specific HRs of nHDL levels for the study outcomes, in which the death events that occur before the study outcome events were censored at the time of the death event. To examine whether the clinical contexts modify the association of nHDL with the study outcomes, we conducted pre-specified subgroup analyses. The subgroups were defined by age, gender, BMI, eGFR, and spot urine albumin-to-creatinine ratio (ACR). Two-sided *p* values < 0.05 were considered statistically significant. Statistical analysis was performed using SPSS for Windows version 22.0 (IBM Corp., Armonk, NY, USA) and R (version 4.1.1; R project for Statistical Computing, Vienna, Austria).

## 3. Results

### 3.1. Baseline Characteristics

To describe the baseline characteristics, the participants were categorized into the tertile by nHDL level (Table 1). The follow-up duration was not significantly different among the three groups. The mean age was highest and lowest in T1 and T3, respectively. The proportion of male gender was not significantly different among the three groups. The proportion with age-adjusted Charlson comorbidity index ≥ 4 was relatively higher in T1. The distribution of the primary cause of CKD, smoking history, and medication history were not significantly different among the three groups. BMI, waist circumference, and SBP and DBP were lowest and highest in T1 and T3, respectively. Hemoglobin, total cholesterol, LDL-C, TG and fasting glucose were also lowest and highest in T1 and T3, respectively. The serum albumin level was highest in T2, while the serum HDL-C level was highest in T1. Kidney function was best preserved in T2, as the spot urine ACR and serum creatinine level were lowest in T2. The echocardiographic indices, except for interventricular wall thickness, were not significantly different among the three groups (Supplementary Materials Table S1). The interventricular wall thickness was significantly increased in T3.

### 3.2. Association of nHDL with Renal Outcomes in Patients with Non-Dialysis CKD

**Figure 2:**
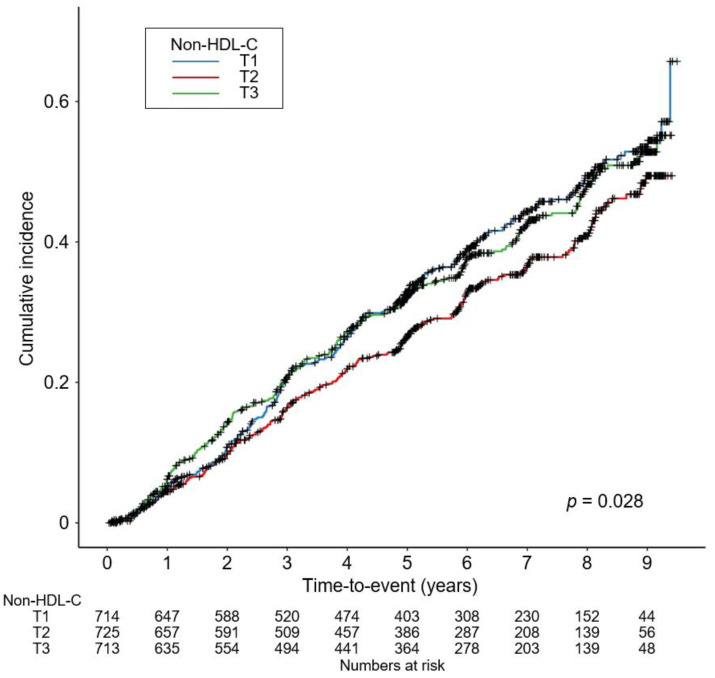
Kaplan–Meier survival curve for cumulative incidence of composite renal event by nHDL. *p* value by Log-rank test. Abbreviations: nHDL, non-high-density lipoprotein cholesterol; T1, 1st tertile; T2, 2nd tertile; T3, 3rd tertile.

**Figure 3:**
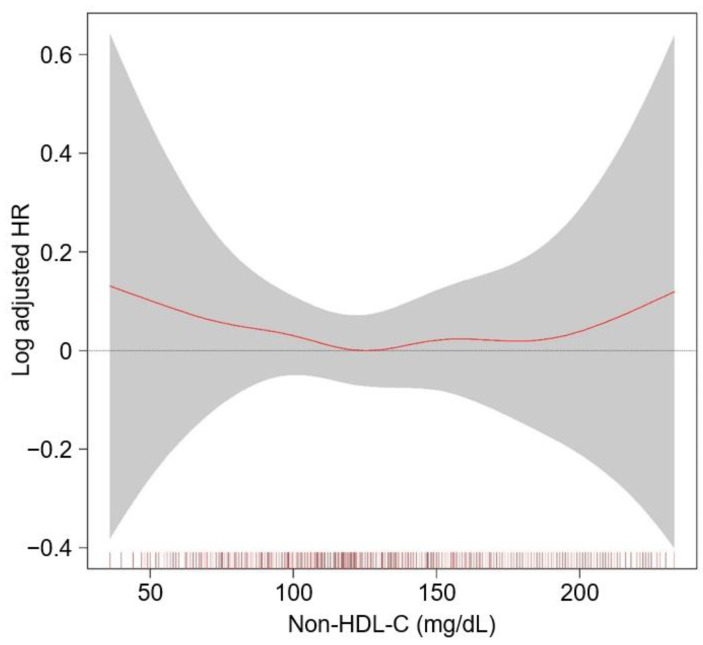
Restricted cubic spline of nHDL on composite renal event. Adjusted HR of nHDL as a continuous variable for a composite CV event is depicted. The model was adjusted for age and sex, age-adjusted Charlson comorbidity index, primary cause of CKD, smoking history, medication (angiotensin-converting enzyme inhibitors/angiotensin receptor blockers, diuretic use, number of antihypertensive drugs, statins), body mass index, waist circumference, systolic blood pressure, hemoglobin, albumin, fasting glucose, hs-CRP, CKD stage, and spot urine ACR. Abbreviations: CI, confidence interval; HR, hazard ratio; nHDL, non-high-density lipoprotein cholesterol; CV, cardiovascular; CKD, chronic kidney disease; hs-CRP, high-sensitivity C-reactive protein; ACR, albumin-to-creatinine ratio.

To visualize the cumulative incidence of the study outcomes by nHDL levels, Kaplan–Meier survival curves were analyzed. The cumulative incidence of a composite renal event was significantly higher in T1 and T3, compared to T2 (*p* = 0.028 by Log-rank test, Figure 2), whereas that of decline of kidney function event (Supplementary Materials Figure S1) or onset of end-stage renal disease (ESRD) event (Supplementary Materials Figure S2) was not significantly different among the groups. To demonstrate an independent association between nHDL and CKD progression, Cox proportional hazard regression models were adopted, where both T1 (adjusted HR (aHR) 1.309, 95% CIs 1.074–1.595) and T3 (aHR 1.272, 95% CIs 1.040–1.556) are associated with significantly increased risk of a composite renal event, compared to T2 (Table 2). The risk of the secondary outcomes was not significantly different among the groups (Table 3). To assess the relation between nHDL (as a continuous variable) with the study outcomes, restricted cubic spline plots were used, which demonstrated a U-shaped association between nHDL and the risk of a composite renal event (Figure 3), whereas the relation of nHDL with the risk of decline of kidney function event (Supplementary Materials Figure S3) or onset of ESRD event (Supplementary Materials Figure S4) was rather linear, but substantially blunted.

### 3.3. Sensitivity Analyses

First, after excluding the subjects with eGFR ≥ 90 mL/min/1.73 m^2^, Cox regression analysis revealed that the risk of primary outcome was still significantly increased both in T1 (aHR 1.290, 95% CIs 1.054–1.579) and T3 (aHR 1.296, 95% CIs 1.055–1.596), compared to T2 (Supplementary Materials Table S2). After excluding the subjects with eGFR ≥ 90 mL/min/1.73 m^2^, the risk of decline of renal function event was not significantly different among the groups, while the risk of initiation of an RRT event was significantly increased in T3, compared to T2 (aHR 1.282, 95% CIs 1.014–1.619). Second, even after excluding the subjects with eGFR < 15 mL/min/1.73 m^2^, the risk of primary outcome was significantly increased both in T1 (aHR 1.369, 95% CIs 1.110–1.688) and T3 (aHR 1.278, 95% CIs 1.031–1.584), compared to T2 (Supplementary Materials Table S3). After excluding the subjects with eGFR < 15 mL/min/1.73 m^2^, the risk of decline of kidney function event was not significantly different among the groups either, whereas the risk of initiation of an RRT event was significantly increased in T3, compared to T2 (aHR 1.332, 95% CIs 1.032–1.719). Finally, in the competing risks analysis to estimate cause-specific HRs, the risk of primary outcome was robustly and significantly higher in T1 (aHR 1.309, 95% CIs 1.051–1.630) and T3 (aHR 1.273, 95% CIs 1.013–1.598), compared to T2, although the risk of the secondary outcomes was not significantly different among the groups (Table 4).

### 3.4. Subgroup Analyses

Subgroup analyses revealed that the association of nHDL with the risk of a composite renal event is altered by gender, BMI, and eGFR (Table 5). More specifically, the risk of a composite renal event was increased only in T3 and T1, compared to that in T2, among the male and female subjects, respectively (*p* for interaction = 0.007). The risk of a composite renal event in T1 and T3 tended to be decreased in the subjects with BMI < 23 kg/m^2^ and eGFR ≥ 45 mL/min/1.73 m^2^, compared to that in T2, whereas the risk in T1 and T3 was significantly increased in the subjects with BMI ≥ 23 kg/m^2^ (*p* for interaction = 0.035) and eGFR < 45 mL/min/1.73 m^2^ (*p* for interaction = 0.011).

## 4. Discussion

In the present study, we found that, contrary to our initial hypothesis, both low and high nHDL are associated with increased risk of CKD progression, demonstrating a non-linear, U-shaped association. We believe that our finding is robust, as the similar results were found in a series of sensitivity analyses, including the analysis of cause-specific hazard models. Moreover, we proved that, in the subgroup analyses, several clinical conditions, such as gender, BMI, and eGFR, alter the association of nHDL with the risk of CKD progression.

It is readily expected that high nHDL increases the risk of CKD progression, which is in agreement with the observation from healthy subjects, where high nHDL significantly increases the risk of incident renal dysfunction [15]. In fact, it has long been suggested that dyslipidemia may promote the development and progression of CKD [25]. Supporting the observations from clinical research, dietary lipid loading aggravates the glomerular lesions in a rodent model of experimental CKD [26]. It has been also reported that elevated levels of very low-density lipoprotein and intermediate-density lipoprotein, which are known to be highly atherogenic, induce proteinuria and glomerulosclerosis in a certain strain of female rats [27]. Therefore, our finding that high nHDL increases the risk of CKD progression seems reasonable.

In contrast, the other finding of the present study that even low nHDL increases the risk of CKD progression is beyond our initial expectation. Based on the definition, either low total cholesterol or a high serum HDL-C level may lead to low nHDL. A low total cholesterol level is closely related to malnutrition and inflammation among the patients with ESRD, leading to mortality [28]. A recent study also reported that those with a high risk of malnutrition were associated with poor baseline kidney function and an increased risk of CKD progression, especially among the elderly [29], suggesting that the association of low nHDL with increased risk of CKD progression in the current study could be attributed to a low total cholesterol level related to the underlying malnutrition-inflammation process.

Meanwhile, low nHDL could result from high serum HDL-C, which is true in the current study (Table 1). It should be noted that, although HDL-C has long been believed as a “good cholesterol” that exerts an anti-inflammatory effect, recent studies report that its anti-inflammatory activity is decreased in patients with CKD [30,31]. Moreover, even the pro-inflammatory role of HDL-C under uremic conditions has been reported [32,33]. Importantly, a study that investigated the association of serum HDL-C level and the risk of CKD progression reported that, not only low, but also high serum HDL-C level increases the risk of CKD progression [14]. Thus, the association between low nHDL and the risk of CKD progression shown in the present study could be directly explained by an elevated serum HDL-C level in patients with CKD.

A previous study reported that high nHDL increases the risk of incident CKD among the healthy male subjects [15], where no significant impact of low nHDL was reported. This may seem somewhat contradictory to our findings, though one should be reminded that the study population is entirely different between the two studies. As our study enrolled only CKD patients at stage 1 to pre-dialysis 5, the biological interpretation of low serum total cholesterol or a high serum HDL-C level should be substantially modified. Indeed, in the subgroup analyses (Table 5), we found that the association of nHDL and the risk of a composite renal event is significantly more prominent in the subjects with eGFR < 45 mL/min/1.73 m^2^, compared to those with eGFR ≥ 45 mL/min/1.73 m^2^.

The use of statins primarily targets a lower serum LDL-C level, though the optimal range of the goal has not been established, due to the lack of firm evidence supporting the benefits of statin therapy on CV outcome in patients with CKD [4,34]. It is of note that the current KDIGO clinical practice guideline does not state the role of LDL-C as well as nHDL in term of renal outcomes, despite accumulating evidence suggesting the detrimental role of dyslipidemia in CKD progression. In this regard, we propose that further studies should determine the optimal target range of nHDL level in patients with CKD.

We acknowledge a number of limitations in the current study. First, we are not able to definitively define the casual relation of nHDL with the risk of CKD progression, due to the observational nature of the present study. Second, the variables measured once at the baseline were included for the regression analyses. Third, as the cohort enrolled only Koreans resident in South Korea, the extrapolation of the data from the current study to the other populations requires a precaution.

## 5. Conclusions

In conclusion, we report that both low and high nHDL are associated with increased risk of CKD progression. Further studies are warranted to determine the optimal target range of nHDL level in patients with CKD.

## Figures and Tables

**Table 1:** Baseline characteristics of study participants by nHDL level.

		nHDL Level		*p* Value
	T1	T2	T3	
Follow-up duration (year)	6.334 ± 2.493	6.320 ± 2.533	6.234 ± 2.538	0.720
Age (year)	55.098 ± 12.452	53.478 ± 11.897	52.620 ± 12.314	0.001
Male	465 (64.1)	434 (60.8)	422 (59.2)	0.144
Age-adjusted CCI				0.001
0–3	330 (45.5)	398 (55.7)	394 (55.3)	
4–5	232 (32.0)	193 (27.0)	207 (29.0)	
6–7	132 (18.2)	105 (14.7)	97 (13.6)	
≥8	31 (4.3)	18 (2.5)	15 (2.1)	
Primary cause of CKD				0.117
DM	215 (29.7)	155 (21.7)	175 (24.6)	
HTN	139 (19.2)	137 (19.2)	150 (21.1)	
GN	211 (29.1)	247 (34.6)	227 (31.9)	
TID	4 (0.6)	5 (0.7)	4 (0.6)	
PKD	107 (14.8)	119 (16.7)	114 (16.0)	
Others	49 (6.8)	51 (7.1)	42 (5.9)	
Smoking history				0.151
Non-smoker	361 (49.8)	395 (55.3)	392 (55.1)	
Ex-smoker	118 (16.3)	108 (15.1)	115 (16.2)	
Current smoker	246 (33.9)	211 (29.6)	204 (28.7)	
Medication				
ACEIs/ARBs	214 (29.5)	197 (27.6)	219 (30.7)	0.425
Diuretic use	237 (32.7)	210 (29.4)	239 (33.5)	0.212
Anti-HTN drugs ≥ 3	214 (29.5)	197 (27.6)	219 (30.7)	0.425
Statins	509 (70.2)	343 (48.0)	266 (37.3)	<0.001
BMI (kg/m^2^)	24.140 ± 3.277	24.583 ± 3.206	25.100 ± 3.681	<0.001
Waist circumference (cm)	86.518 ± 9.543	87.410 ± 9.470	88.659 ± 10.089	<0.001
SBP (mmHg)	125.552 ± 15.070	128.585 ± 16.716	129.361 ± 16.530	<0.001
DBP (mmHg)	74.724 ± 10.425	77.742 ± 10.952	78.303 ± 11.567	<0.001
Laboratory findings				
Hemoglobin (g/dL)	12.520 ± 2.019	12.970 ± 2.000	13.031 ± 2.010	<0.001
Albumin (g/dL)	4.188 ± 0.353	4.205 ± 0.371	4.132 ± 0.530	0.010
Total cholesterol (mg/dL)	138.422 ± 21.093	169.807 ± 16.377	214.798 ± 30.740	<0.001
HDL-C (mg/dL)	50.692 ± 17.316	48.418 ± 14.229	48.592 ± 14.407	0.013
LDL-C (mg/dL)	68.828 ± 15.342	94.705 ± 16.829	127.746 ± 27.669	<0.001
TG (mg/dL)	117.327 ± 58.870	152.076 ± 77.792	204.278 ± 125.992	<0.001
Fasting glucose (mg/dL)	107.427 ± 33.661	110.925 ± 41.189	115.025 ± 44.121	0.001
hs-CRP (mg/dL)	0.500 (0.200, 1.400)	0.600 (0.210, 1.700)	0.800 (0.300, 1.900)	0.449
Spot urine ACR (mg/g)	331.017 (80.954, 891.067)	312.364 (63.343, 950.315)	426.526 (91.296, 1489.556)	<0.001
Creatinine (mg/dL)	1.925 ± 1.220	1.768 ± 1.133	1.767 ± 1.103	0.015
eGFR (mL/min/1.73 m^2^)	47.145 ± 28.686	52.215 ± 30.834	51.965 ± 30.823	0.001
CKD stages				0.111
Stage 1	93 (12.8)	130 (18.2)	125 (17.5)	
Stage 2	132 (18.2)	135 (18.9)	140 (19.6)	
Stage 3a	114 (15.7)	117 (16.4)	122 (17.1)	
Stage 3b	161 (22.2)	149 (20.9)	143 (20.1)	
Stage 4	169 (23.3)	146 (20.4)	141 (19.8)	
Stage 5	56 (7.7)	37 (5.2)	42 (5.9)	

Values for categorical variables are given as a number (percentage); values for continuous variables, as mean ± standard deviation or median (interquartile range). Abbreviations: ACEIs, angiotensin converting enzyme inhibitors; ACR, albumin-to-creatinine ratio; ARBs, angiotensin receptor blockers; BMI, body mass index; CCI, Charlson comorbidity index; CKD, chronic kidney disease; DBP, diastolic blood pressure; DM, diabetes mellitus; eGFR, estimated glomerular filtration rate; GN, glomerulonephritis; hs-CRP, high-sensitivity C-reactive protein; HTN, hypertension; LDL-C, low-density lipoprotein cholesterol; nHDL, non-high-density lipoprotein cholesterol; PKD, polycystic kidney disease; SBP, systolic blood pressure; T1, 1st tertile; T2, 2nd tertile; T3, 3rd tertile; TG, triglyceride; TID, tubulointerstitial disease; HDL-C, high-density lipoprotein cholesterol.

**Table 2:** HRs for the primary outcome by nHDL level.

	NHDL	Events, *n* (%)	Model 1		Model 2		Model 3		Model 4	
			HR (95% CIs)	*p* Value	HR (95% CIs)	*p* Value	HR (95% CIs)	*p* Value	HR (95% CIs)	*p* Value
Composite renal event	T1	295 (40.7)	1.302 (1.084, 1.564)	0.005	1.224 (1.033, 1.452)	0.020	1.178 (0.982, 1.414)	0.077	1.309 (1.074, 1.595)	0.008
	T2	243 (34.0)	Reference		Reference		Reference		Reference	
	T3	278 (39.0)	1.207 (1.000, 1.456)	0.050	1.213 (1.021, 1.440)	0.028	1.216 (1.015, 1.457)	0.033	1.272 (1.040, 1.556)	0.019

Model 1, unadjusted model. Model 2, model 1 + adjusted for age and sex. Model 3, model 2 + adjusted for age-adjusted Charlson comorbidity index, primary cause of CKD, smoking history, medication (angiotensin-converting enzyme inhibitors/angiotensin receptor blockers, diuretic use, number of antihypertensive drugs, statins), body mass index, waist circumference, and systolic blood pressure. Model 4, model 3 + adjusted for hemoglobin, albumin, fasting glucose, hs-CRP, CKD stage, and spot urine ACR. Abbreviations: CI, confidence interval; HR, hazard ratio; nHDL, non-high-density lipoprotein cholesterol; T1, 1st tertile; T2, 2nd tertile; T3, 3rd tertile.

**Table 3:** HRs for the secondary outcomes by nHDL level.

	NHDL	Events, *n* (%)	Model 1		Model 2		Model 3		Model 4	
			HR (95% CIs)	*p* Value	HR (95% CIs)	*p* Value	HR (95% CIs)	*p* Value	HR (95% CIs)	*p* Value
Decline of renal function	T1	198 (27.3)	1.162 (0.932, 1.448)	0.182	1.144 (0.933, 1.403)	0.197	1.055 (0.846, 1.316)	0.634	1.110 (0.882, 1.398)	0.373
	T2	174 (24.4)	Reference		Reference		Reference		Reference	
	T3	197 (27.6)	1.148 (0.917, 1.436)	0.228	1.193 (0.973, 1.463)	0.089	1.209 (0.976, 1.498)	0.082	1.002 (0.791, 1.271)	0.984
Initiation of RRT	T1	226 (31.2)	1.293 (1.047, 1.597)	0.017	1.207 (0.993, 1.468)	0.058	1.139 (0.924, 1.404)	0.224	1.151 (0.915, 1.449)	0.231
	T2	184 (25.8)	Reference		Reference		Reference		Reference	
	T3	214 (30.0)	1.247 (1.005, 1.546)	0.045	1.228 (1.008, 1.495)	0.041	1.224 (0.995, 1.506)	0.056	1.281 (1.014, 1.619)	0.038

Model 1, unadjusted model. Model 2, model 1 + adjusted for age and sex. Model 3, model 2 + adjusted for age-adjusted Charlson comorbidity index, primary cause of CKD, smoking history, medication (angiotensin-converting enzyme inhibitors/angiotensin receptor blockers, diuretic use, number of antihypertensive drugs, statins), body mass index, waist circumference, and systolic blood pressure. Model 4, model 3 + adjusted for hemoglobin, albumin, fasting glucose, hs-CRP, CKD stage, and spot urine ACR. Abbreviations: CI, confidence interval; HR, hazard ratio; nHDL, non-high-density lipoprotein cholesterol; RRT, renal replacement therapy; T1, 1st tertile; T2, 2nd tertile; T3, 3rd tertile.

**Table 4:** Cause-specific HRs for the study outcomes by nHDL level.

	NHDL	Model 1		Model 2		Model 3		Model 4	
		HR (95% CIs)	*p* value	HR (95% CIs)	*p* value	HR (95% CIs)	*p* value	HR (95% CIs)	*p* value
Composite renal event	T1	1.240 (1.047, 1.467)	0.013	1.224 (1.033, 1.451)	0.019	1.178 (0.976, 1.432)	0.088	1.309 (1.051, 1.630)	0.016
	T2	Reference		Reference		Reference		Reference	
	T3	1.209 (1.018, 1.437)	0.031	1.213 (1.021, 1.441)	0.028	1.216 (1.010, 1.464)	0.039	1.273 (1.013, 1.598)	0.038
Decline renal function	T1	1.139 (0.930, 1.395)	0.207	1.144 (0.933, 1.402)	0.196	1.055 (0.843, 1.321)	0.640	1.111 (0.883, 1.396)	0.369
	T2	Reference		Reference		Reference		Reference	
	T3	1.195 (0.974, 1.466)	0.087	1.193 (0.973, 1.464)	0.090	1.209 (0.972, 1.504)	0.088	1.002 (0.780, 1.288)	0.985
Initiation of RRT	T1	1.236 (1.018, 1.501)	0.032	1.207 (0.993, 1.468)	0.059	1.139 (0.915, 1.417)	0.243	1.151 (0.887, 1.484)	0.290
	T2	Reference		Reference		Reference		Reference	
	T3	1.219 (1.001, 1.485)	0.049	1.228 (1.008, 1.495)	0.041	1.224 (0.991, 1.513)	0.061	1.282 (0.977, 1.683)	0.073

Model 1, unadjusted model. Model 2, model 1 + adjusted for age and sex. Model 3, model 2 + adjusted for age-adjusted Charlson comorbidity index, primary cause of CKD, smoking history, medication (angiotensin-converting enzyme inhibitors/angiotensin receptor blockers, diuretic use, number of antihypertensive drugs, statins), body mass index, waist circumference, and systolic blood pressure. Model 4, model 3 + adjusted for hemoglobin, albumin, fasting glucose, hs-CRP, CKD stage, and spot urine ACR. Abbreviations: CI, confidence interval; HR, hazard ratio; nHDL, non-high-density lipoprotein cholesterol; RRT, renal replacement therapy; T1, 1st tertile; T2, 2nd tertile, T3, 3rd tertile.

**Table 5:** HRs for the primary outcome by nHDL level in various subgroups.

	NHDL	Events, *n* (%)	Unadjusted HR (95% CIs)	*p* for Interaction	Adjusted HR (95% CIs)	*p* for Interaction
Age < 60 years	T1	173 (40.7)	1.255 (1.011, 1.556)	0.720	1.256 (0.972, 1.624)	0.157
	T2	159 (33.3)	Reference		Reference	
	T3	188 (39.0)	1.270 (1.028, 1.569)		1.320 (1.024, 1.701)	
Age ≥ 60 years	T1	122 (40.7)	1.168 (0.885, 1.543)		1.396 (1.010, 1.929)	
	T2	84 (35.6)	Reference		Reference	
	T3	90 (39.0)	1.087 (0.807, 1.464)		1.1685, 0.817, 1.670)	
Male	T1	174 (37.4)	1.084 (0.872, 1.348)	0.153	1.047 (0.807, 1.359)	0.007
	T2	151 (34.8)	Reference		Reference	
	T3	162 (38.4)	1.145 (0.917, 1.430)		1.453 (1.114, 1.895)	
Female	T1	121 (46.5)	1.528 (1.165, 2.004)		1.506 (1.086, 2.088)	
	T2	92 (32.9)	Reference		Reference	
	T3	116 (39.9)	1.315 (1.000, 1.729)		0.993 (0.710, 1.388)	
BMI < 23 kg/m^2^	T1	111 (41.4)	1.252 (0.936, 1.675)	0.898	0.794 (0.558, 1.129)	0.035
	T2	77 (34.7)	Reference		Reference	
	T3	82 (39.6)	1.289 (0.944, 1.760)		0.980 (0.673, 1.425)	
BMI ≥ 23 kg/m^2^	T1	183 (40.6)	1.233 (0.999, 1.522)		1.562 (1.217, 2.005)	
	T2	164 (33.7)	Reference		Reference	
	T3	196 (39.0)	1.186 (0.963, 1.459)		1.433 (1.120, 1.835)	
eGFR ≥ 45 mL/min/1.73 m^2^	T1	51 (16.2)	1.036 (0.707, 1.520)	0.585	0.994 (0.651, 1.518)	0.011
	T2	54 (14.8)	Reference		Reference	
	T3	60 (16.6)	1.094 (0.757, 1.580)		0.736 (0.478, 1.133)	
eGFR < 45 mL/min/1.73 m^2^	T1	244 (59.5)	1.183 (0.978, 1.431)		1.313 (1.046, 1.648)	
	T2	189 (54.2)	Reference		Reference	
	T3	218 (62.1)	1.353 (1.113, 1.644)		1.426 (1.131, 1.797)	
Spot urine ACR < 300 mg/g	T1	88 (26.4)	1.486 (1.073, 2.057)	0.173	1.400 (0.951, 2.059)	0.800
	T2	62 (18.4)	Reference		Reference	
	T3	57 (19.6)	1.031 (0.720, 1.478)		1.229 (0.824, 1.833)	
Spot urine ACR ≥ 300 mg/g	T1	202 (55.5)	1.157 (0.945, 1.416)		1.259 (0.993, 1.593)	
	T2	176 (49.9)	Reference		Reference	
	T3	213 (53.4)	1.161 (0.951, 1.418)		1.290 (1.015, 1.639)	

The model was adjusted for age and sex, age-adjusted Charlson comorbidity index, primary cause of CKD, smoking history, medication (angiotensin-converting enzyme inhibitors/angiotensin receptor blocker, diuretic use, number of antihypertensive drugs, statins), body mass index, waist circumference, systolic blood pressure, hemoglobin, albumin, fasting glucose, hs-CRP, CKD stage, and spot urine ACR. Abbreviations: CI, confidence interval; eGFR, estimated glomerular filtration rate; HR, hazard ratio; nHDL, non-high-density lipoprotein cholesterol; T1, 1st tertile; T2, 2nd tertile; T3, 3rd tertile.

## Data Availability

Not applicable.

## Supplementary Materials

The following are available online at https://www.mdpi.com/article/10.3390/nu14214704/s1, Figure S1: Kaplan–Meier survival curve for cumulative incidence of decline of kidney function by nHDL; Figure S2: Kaplan–Meier survival curve for cumulative incidence of onset of ESRD by nHDL; Figure S3: Restricted cubic spline of nHDL on decline of kidney function; Figure S4: Restricted cubic spline of nHDL on onset of ESRD; Table S1: Summary of echocardiographic findings of study participants by nHDL; Table S2: HRs for the primary and secondary outcomes by nHDL level after excluding the subjects at CKD stage 1; Table S3: HRs for the primary and secondary outcomes by nHDL level after excluding the subjects at CKD stage 5.

## References

[1] Tsuruya K., Yoshida H., Nagata M., Kitazono T., Iseki K., Iseki C., Fujimoto S., Konta T., Moriyama T., Yamagata K. (2015). Impact of the Triglycerides to High-Density Lipoprotein Cholesterol Ratio on the Incidence and Progression of CKD: A Longitudinal Study in a Large Japanese Population. Am. J. Kidney Dis..

[2] Kochan Z., Szupryczynska N., Malgorzewicz S., Karbowska J. (2021). Dietary Lipids and Dyslipidemia in Chronic Kidney Disease. Nutrients.

[3] Tsuruya K., Yoshida H., Nagata M., Kitazono T., Iseki K., Iseki C., Fujimoto S., Konta T., Moriyama T., Yamagata K. (2017). Association of Hypertriglyceridemia With the Incidence and Progression of Chronic Kidney Disease and Modification of the Association by Daily Alcohol Consumption. J. Ren. Nutr. Off. J. Counc. Ren. Nutr. Natl. Kidney Found..

[4] Wanner C., Tonelli M. (2014). KDIGO Clinical Practice Guideline for Lipid Management in CKD: Summary of recommendation statements and clinical approach to the patient. Kidney Int..

[5] Toth P.P., Philip S., Hull M., Granowitz C. (2019). Association of Elevated Triglycerides With Increased Cardiovascular Risk and Direct Costs in Statin-Treated Patients. Mayo Clin. Proc..

[6] Nelson A.J., Navar A.M., Mulder H., Wojdyla D., Philip S., Granowitz C., Peterson E.D., Pagidipati N.J. (2020). Association Between Triglycerides and Residual Cardiovascular Risk in Patients With Type 2 Diabetes Mellitus and Established Cardiovascular Disease (From the Bypass Angioplasty Revascularization Investigation 2 Diabetes [BARI 2D] Trial). Am. J. Cardiol..

[7] Li Y.H., Tseng W.K., Yin W.H., Lin F.J., Wu Y.W., Hsieh I.C., Lin T.H., Sheu W.H., Yeh H.I., Chen J.W. (2020). Prognostic effect of high-density lipoprotein cholesterol level in patients with atherosclerotic cardiovascular disease under statin treatment. Sci. Rep..

[8] Sonmez A., Yilmaz M.I., Saglam M., Unal H.U., Gok M., Cetinkaya H., Karaman M., Haymana C., Eyileten T., Oguz Y. (2015). The role of plasma triglyceride/high-density lipoprotein cholesterol ratio to predict cardiovascular outcomes in chronic kidney disease. Lipids Health Dis..

[9] Kim J.Y., Park J.T., Kim H.W., Chang T.I., Kang E.W., Ahn C., Oh K.H., Lee J., Chung W., Kim Y.S. (2021). Inflammation Alters Relationship Between High-Density Lipoprotein Cholesterol and Cardiovascular Risk in Patients With Chronic Kidney Disease: Results From KNOW-CKD. J. Am. Heart Assoc..

[10] Kang H.T., Kim J.K., Kim J.Y., Linton J.A., Yoon J.H., Koh S.B. (2012). Independent association of TG/HDL-C with urinary albumin excretion in normotensive subjects in a rural Korean population. Clin. Chim. Acta Int. J. Clin. Chem..

[11] Kang H.T., Shim J.Y., Lee Y.J., Lee J.E., Linton J.A., Kim J.K., Lee H.R. (2011). Association between the ratio of triglycerides to high-density lipoprotein cholesterol and chronic kidney disease in Korean adults: The 2005 Korean National Health and Nutrition Examination Survey. Kidney Blood Press. Res..

[12] Kim J.Y., Kang H.T., Lee H.R., Lee Y.J., Shim J.Y. (2012). Comparison of lipid-related ratios for prediction of chronic kidney disease stage 3 or more in Korean adults. J. Korean Med. Sci..

[13] Tsuruya K., Yoshida H., Nagata M., Kitazono T., Hirakata H., Iseki K., Moriyama T., Yamagata K., Yoshida H., Fujimoto S. (2014). Association of the triglycerides to high-density lipoprotein cholesterol ratio with the risk of chronic kidney disease: Analysis in a large Japanese population. Atherosclerosis.

[14] Nam K.H., Chang T.I., Joo Y.S., Kim J., Lee S., Lee C., Yun H.R., Park J.T., Yoo T.H., Sung S.A. (2019). Association Between Serum High-Density Lipoprotein Cholesterol Levels and Progression of Chronic Kidney Disease: Results From the KNOW-CKD. J. Am. Heart Assoc..

[15] Schaeffner E.S., Kurth T., Curhan G.C., Glynn R.J., Rexrode K.M., Baigent C., Buring J.E., Gaziano J.M. (2003). Cholesterol and the risk of renal dysfunction in apparently healthy men. J. Am. Soc. Nephrol..

[16] Muntner P., Coresh J., Smith J.C., Eckfeldt J., Klag M.J. (2000). Plasma lipids and risk of developing renal dysfunction: The atherosclerosis risk in communities study. Kidney Int..

[17] Fox C.S., Larson M.G., Leip E.P., Culleton B., Wilson P.W., Levy D. (2004). Predictors of new-onset kidney disease in a community-based population. JAMA.

[18] Executive Summary of The Third Report of The National Cholesterol Education Program (NCEP) (2001). Expert Panel on Detection, Evaluation, And Treatment of High Blood Cholesterol In Adults (Adult Treatment Panel III). JAMA.

[19] Su X., Kong Y., Peng D. (2019). Evidence for changing lipid management strategy to focus on non-high density lipoprotein cholesterol. Lipids Health Dis..

[20] Suh S.H., Oh T.R., Choi H.S., Kim C.S., Bae E.H., Ma S.K., Oh K.-H., Han S.H., Kim S.W. (2022). Non-High-Density Lipoprotein Cholesterol and Cardiovascular Outcomes in Chronic Kidney Disease: Results from KNOW-CKD Study. Nutrients.

[21] Oh K.H., Park S.K., Park H.C., Chin H.J., Chae D.W., Choi K.H., Han S.H., Yoo T.H., Lee K., Kim Y.S. (2014). KNOW-CKD (KoreaN cohort study for Outcome in patients With Chronic Kidney Disease): Design and methods. BMC Nephrol..

[22] Levey A.S., Stevens L.A., Schmid C.H., Zhang Y.L., Castro A.F., Feldman H.I., Kusek J.W., Eggers P., Van Lente F., Greene T. (2009). A new equation to estimate glomerular filtration rate. Ann. Intern. Med..

[23] Levey A.S., Eckardt K.U., Tsukamoto Y., Levin A., Coresh J., Rossert J., De Zeeuw D., Hostetter T.H., Lameire N., Eknoyan G. (2005). Definition and classification of chronic kidney disease: A position statement from Kidney Disease: Improving Global Outcomes (KDIGO). Kidney Int..

[24] Lang R.M., Bierig M., Devereux R.B., Flachskampf F.A., Foster E., Pellikka P.A., Picard M.H., Roman M.J., Seward J., Shanewise J.S. (2005). Recommendations for chamber quantification: A report from the American Society of Echocardiography’s Guidelines and Standards Committee and the Chamber Quantification Writing Group, developed in conjunction with the European Association of Echocardiography, a branch of the European Society of Cardiology. J. Am. Soc. Echocardiogr..

[25] Moorhead J.F., Chan M.K., El-Nahas M., Varghese Z. (1982). Lipid nephrotoxicity in chronic progressive glomerular and tubulo-interstitial disease. Lancet.

[26] Gröne H.J., Walli A., Gröne E., Niedmann P., Thiery J., Seidel D., Helmchen U. (1989). Induction of glomerulosclerosis by dietary lipids. A functional and morphologic study in the rat. Lab. Investig. A J. Tech. Methods Pathol..

[27] Joles J.A., van Goor H., van der Horst M.L., van Tol A., Elema J.D., Koomans H.A. (1995). High lipid levels in very low density lipoprotein and intermediate density lipoprotein may cause proteinuria and glomerulosclerosis in aging female analbuminemic rats. Lab. Investig. A J. Tech. Methods Pathol..

[28] Liu Y., Coresh J., Eustace J.A., Longenecker J.C., Jaar B., Fink N.E., Tracy R.P., Powe N.R., Klag M.J. (2004). Association between cholesterol level and mortality in dialysis patients: Role of inflammation and malnutrition. JAMA.

[29] Lu Y., Nyunt M.S.Z., Gao Q., Gwee X., Chua D.Q., Yap K.B., Pan F., Ng T.P. (2022). Malnutrition Risk and Kidney Function and Decline in Community-Dwelling Older Adults. J. Ren. Nutr..

[30] Gluba-Brzozka A., Franczyk B., Rysz J. (2019). Cholesterol Disturbances and the Role of Proper Nutrition in CKD Patients. Nutrients.

[31] Vaziri N.D., Navab M., Fogelman A.M. (2010). HDL metabolism and activity in chronic kidney disease. Nat. Rev. Nephrol..

[32] Yamamoto S., Yancey P.G., Ikizler T.A., Jerome W.G., Kaseda R., Cox B., Bian A., Shintani A., Fogo A.B., Linton M.F. (2012). Dysfunctional high-density lipoprotein in patients on chronic hemodialysis. J. Am. Coll. Cardiol..

[33] Moradi H., Vaziri N.D., Kashyap M.L., Said H.M., Kalantar-Zadeh K. (2013). Role of HDL dysfunction in end-stage renal disease: A double-edged sword. J. Ren. Nutr..

[34] Baigent C., Landray M.J., Reith C., Emberson J., Wheeler D.C., Tomson C., Wanner C., Krane V., Cass A., Craig J. (2011). The effects of lowering LDL cholesterol with simvastatin plus ezetimibe in patients with chronic kidney disease (Study of Heart and Renal Protection): A randomised placebo-controlled trial. Lancet.

