# Supplementary material for: Non-High-Density Lipoprotein Cholesterol and Progression of Chronic Kidney Disease: Results from the KNOW-CKD Study
Source: Nutrients. 2022 Nov 7;14(21):4704. doi: 10.3390/nu14214704 (PMC9656579; doi:10.3390/nu14214704)
Supplement: Supplementary file 1 [file nutrients-14-04704-s001.zip › nutrients-1992275-supplementary.pdf]

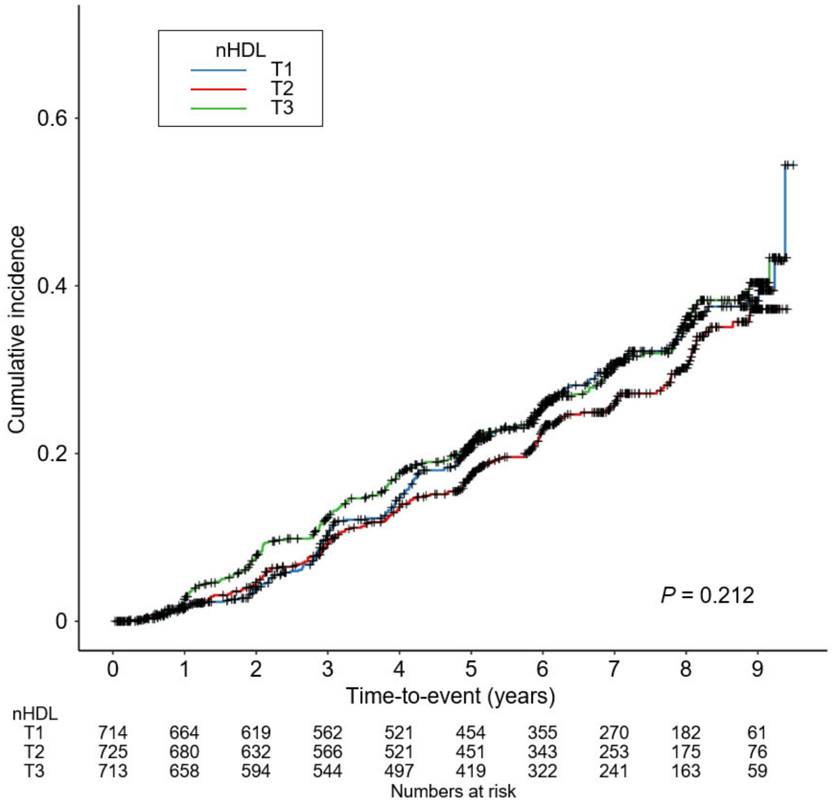

Figure S1. Kaplan-Meier survival curve for cumulative incidence of decline of kidney function by nHDL. Note: *P* value by Log-rank test. Abbreviations: nHDL, non-high density lipoprotein cholesterol; T1, 1st tertile; T2, 2nd tertile, T3, 3rd tertile.

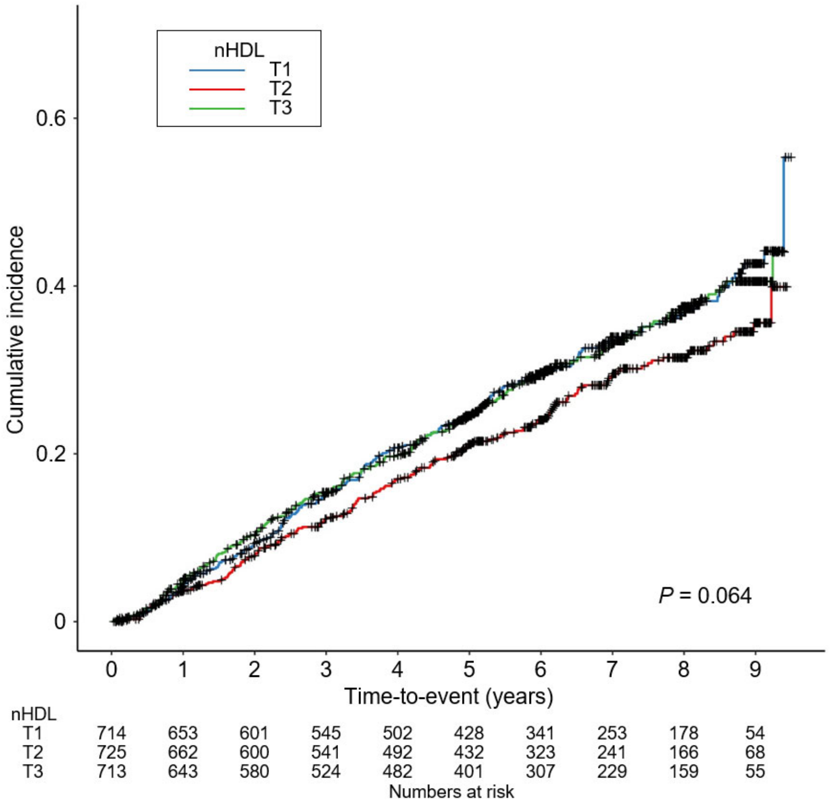

Figure S2. Kaplan-Meier survival curve for cumulative incidence of onset of ESRD by nHDL. Note: *P* value by Log-rank test. Abbreviations: nHDL, non-high density lipoprotein cholesterol; T1, 1st tertile; T2, 2nd tertile, T3, 3rd tertile; ESRD, end-stage renal disease.

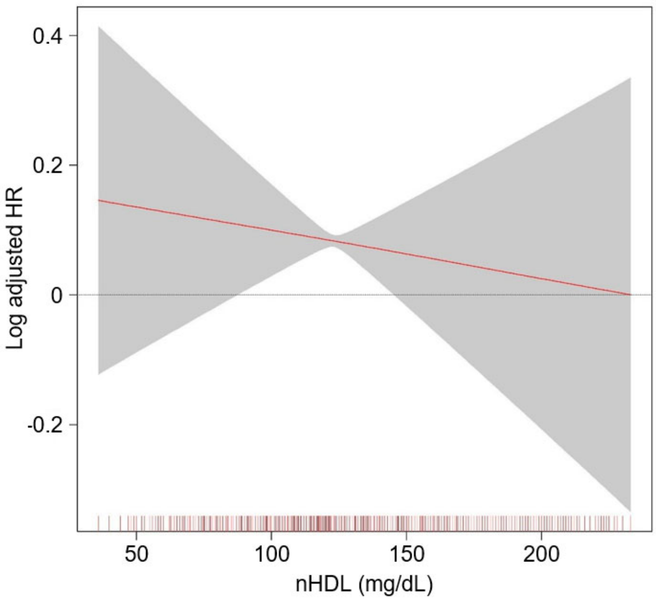

Figure S3. Restricted cubic spline of nHDL on decline of kidney function. Note: Adjusted HR of nHDL as a continuous variable for composite CV event is depicted. The model was adjusted for age and sex, age-, statins), BMI, WC, SBP, hemoglobin, albumin, fasting glucose, hs-CRP, CKD stage, and spot urine ACR. Abbreviations: CI, confidence interval; HR, hazard ratio; nHDL, non-high density lipoprotein cholesterol; CV, cardiovascular; BMI, body mass index; WC, waist circumference; SBP, systolic blood pressure; hs-CRP, high-sensitivity C-reactive protein; CKD, chronic kidney disease; ACR, albumin-to-creatinine ratio.

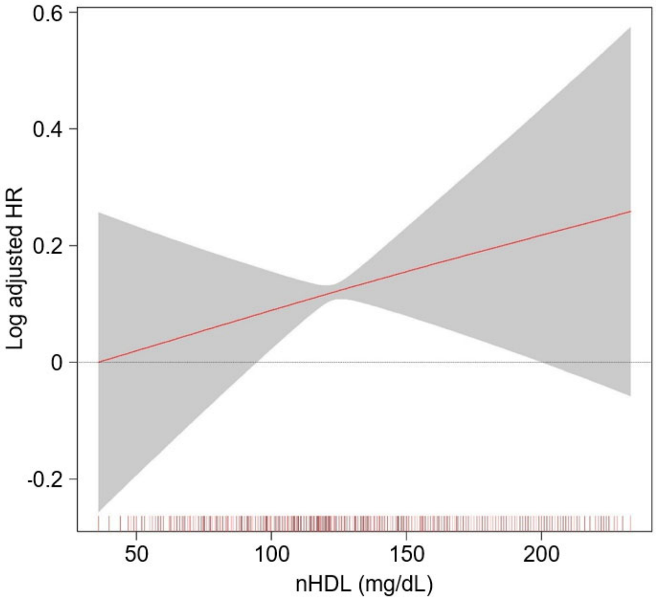

Figure S4. Restricted cubic spline of nHDL on onset of ESRD. Note: Adjusted HR of nHDL as a continuous variable for composite CV event is depicted. The model was adjusted for age and sex, age-adjusted Charlson comorbidity index, primary renal disease, smoking status, medication (ACEIs/ARBs, diuretics, number of anti-HTN drugs, statins), BMI, WC, SBP, hemoglobin, albumin, fasting glucose, hs-CRP, CKD stage, and spot urine ACR. Abbreviations: CI, confidence interval; HR, hazard ratio; nHDL, non-high density lipoprotein cholesterol; ACEIs, angiotensin-converting enzyme inhibitors; ARBs, angiotensin receptor blockers; HTN, hypertension.

Table S1. Summary of echocardiographic findings of study participants by nHDL.

	nHDL			
	**T1**	**T2**	**T3**	***P* value**
LVMI (g/m^2)	93.581 ± 25.051	92.959 ± 24.155	95.082 ± 26.570	0.303
E/e′	9.960 ± 3.547	9.882 ± 4.180	10.087 ± 3.985	0.638
LVEF (%)	63.779 ± 6.304	64.188 ± 6.479	64.275 ± 6.148	0.283
LAD (mm)	38.206 ± 6.086	37.689 ± 5.824	37.727 ± 5.692	0.196
RWMA	28 (3.9)	19 (2.7)	19 (2.7)	0.601
Valve calcification	73 (10.2)	58 (8.2)	55 (7.9)	0.238
PWT (mm)	9.247 ± 1.606	9.228 ± 1.527	9.366 ± 1.628	0.217
IVWT (mm)	9.277 ± 1.724	9.291 ± 1.622	9.561 ± 1.817	0.003
LVEDD (mm)	48.815 ± 4.640	48.785 ± 4.386	48.432 ± 4.504	0.209
LVESD (mm)	30.487 ± 4.267	30.486 ± 4.207	30.231 ± 4.366	0.443

Note: Values for categorical variables are given as number (percentage); values for continuous variables, as mean ± standard deviation or median (interquartile range). Abbreviations: E/e′, ratio of the early transmitral blood flow velocity to early diastolic velocity of the mitral annulus; nHDL-C, non-high density lipoprotein cholesterol; IVWT, interventricular wall thickness; LAD, left atrium diameter; LVEDD, left ventricular end-diastolic diameter; LVEF, left ventricular ejection fraction; LVESD, left ventricular end-systolic diameter; LVMI, left ventricular mass index; nHDL, non-high density lipoprotein cholesterol; PWT, posterior wall thickness; RMWA, regional wall motion abnormality; T1, 1st tertile; T2, 2nd tertile; T3, 3rd tertile.

.

Table S2. HRs for the primary and secondary outcomes by nHDL level after excluding the subjects at CKD stage 1.

	nHDL	Events, *n* (%)	Model 1		Model 2		Model 3		Model 4	
			HR (95% CIs)	*P* value	HR (95% CIs)	*P* value	HR (95% CIs)	*P* value	HR (95% CIs)	*P* value
Composite renal event	T1	286 (45.3)	1.197 (0.993, 1.442)	0.059	1.175 (0.989, 1.397)	0.067	1.146 (0.952, 1.380)	0.151	1.290 (1.054, 1.579)	0.014
	T2	235 (40.2)	Reference		Reference		Reference		Reference	
	T3	271 (46.1)	1.222 (1.009, 1.479)	0.041	1.209 (1.015, 1.440)	0.033	1.237 (1.030, 1.486)	0.023	1.296 (1.055, 1.592)	0.014
Decline renal function	T1	190 (30.1)	1.078 (0.859, 1.351)	0.518	1.098 (0.891, 1.352)	0.380	1.021 (0.813, 1.281)	0.859	1.091 (0.861, 1.383)	0.469
	T2	166 (28.4)	Reference		Reference		Reference		Reference	
	T3	190 (32.3)	1.159 (0.921, 1.459)	0.208	1.194 (0.969, 1.471)	0.096	1.215 (0.976, 1.513)	0.081	1.008 (0.790, 1.286)	0.950
Initiation of RRT	T1	224 (35.4)	1.169 (0.946, 1.445)	0.147	1.143 (0.940, 1.390)	0.179	1.099 (0.891, 1.357)	0.377	1.137 (0.903, 1.431)	0.276
	T2	184 (31.5)	Reference		Reference		Reference		Reference	
	T3	214 (36.4)	1.252 (1.009, 1.553)	0.041	1.214 (0.997, 1.479)	0.054	1.232 (1.002, 1.516)	0.048	1.282 (1.014, 1.619)	0.038

Note: Model 1, unadjusted model. Model 2, model 1 + adjusted for age and sex. Model 3, model 2 + adjusted for age-adjusted Charlson comorbidity index, primary renal disease, smoking status, medication (ACEIs/ARBs, diuretics, number of anti-HTN drugs, statins), BMI, WC, and SBP. Model 4, model 3 + adjusted for hemoglobin, albumin, fasting glucose, hs-CRP, CKD stage, and spot urine ACR. Abbreviations: CI, confidence interval; HR, hazard ratio; nHDL, non-high density lipoprotein cholesterol; RRT, renal replacement therapy; T1, 1st tertile; T2, 2nd tertile, T3, 3rd tertile; ACEIs, angiotensin-converting enzyme inhibitors; ARBs, angiotensin receptor blockers; HTN, hypertension; BMI, body mass index; WC, waist circumference; SBP, systolic blood pressure; hs-CRP, high-sensitivity C-reactive protein; CKD, chronic kidney disease; ACR, albumin-to-creatinine ratio.

Table S3. HRs for the primary and secondary outcomes by nHDL level after excluding the subjects at CKD stage 5.

	nHDL	Events, *n* (%)	Model 1		Model 2		Model 3		Model 4	
			HR (95% CIs)	*P* value	HR (95% CIs)	*P* value	HR (95% CIs)	*P* value	HR (95% CIs)	*P* value
Composite renal event	T1	251 (37.5)	1.300 (1.066, 1.584)	0.010	1.220 (1.015, 1.466)	0.034	1.162 (0.954, 1.415)	0.135	1.369 (1.110, 1.688)	0.003
	T2	210 (31.0)	Reference		Reference		Reference		Reference	
	T3	243 (36.2)	1.226 (1.002, 1.501)	0.048	1.232 (1.024, 1.482)	0.027	1.255 (1.034, 1.523)	0.021	1.278 (1.031, 1.584)	0.025
Decline renal function	T1	191 (28.6)	1.180 (0.943, 1.478)	0.148	1.166 (0.946, 1.436)	0.149	1.072 (0.856, 1.342)	0.545	1.129 (0.893, 1.427)	0.309
	T2	166 (24.5)	Reference		Reference		Reference		Reference	
	T3	187 (27.9)	1.137 (0.904, 1.430)	0.272	1.184 (0.961, 1.459)	0.113	1.183 (0.950, 1.473)	0.133	0.974 (0.764, 1.242)	0.833
Initiation of RRT	T1	182 (27.2)	1.284 (1.015, 1.625)	0.037	1.196 (0.963, 1.485)	0.105	1.111 (0.880, 1.402)	0.378	1.194 (0.930, 1.533)	0.165
	T2	151 (22.3)	Reference		Reference		Reference		Reference	
	T3	181 (27.0)	1.296 (1.022, 1.643)	0.032	1.272 (1.025, 1.579)	0.029	1.301 (1.036, 1.633)	0.024	1.332 (1.032, 1.719)	0.028

Note: Model 1, unadjusted model. Model 2, model 1 + adjusted for age and sex. Model 3, model 2 + adjusted for age-adjusted Charlson comorbidity index, primary renal disease, smoking status, medication (ACEIs/ARBs, diuretics, number of anti-HTN drugs, statins), BMI, WC, and SBP. Model 4, model 3 + adjusted for hemoglobin, albumin, fasting glucose, hs-CRP, CKD stage, and spot urine ACR. Abbreviations: CI, confidence interval; HR, hazard ratio; nHDL, non-high density lipoprotein cholesterol; RRT, renal replacement therapy; T1, 1st tertile; T2, 2nd tertile, T3, 3rd tertile.
